# Effects of Bitumen Thickness on the Aging Behavior of High-Content Polymer-Modified Asphalt Mixture

**DOI:** 10.3390/polym15102325

**Published:** 2023-05-16

**Authors:** Peng Lin, Xueyan Liu, Shisong Ren, Jian Xu, Yi Li, Mingliang Li

**Affiliations:** 1 Key Laboratory of Transport Industry of Road Structure and Material, Research Institute of Highway, Ministry of Transport, Xitucheng Road No. 8, Beijing 100088, China; 2 Section of Pavement Engineering, Faculty of Civil Engineering & Geosciences, Delft University of Technology, Stevinweg 1, 2628 CN Delft, The Netherlands

**Keywords:** film thickness, HCPMA, optimal film thickness, aging durability

## Abstract

The film thickness of asphalt mixtures is critical for determining their performance and aging durability. However, understanding of the appropriate film thickness and its influence on performance and aging behavior for high-content polymer-modified asphalt (HCPMA) mixtures is still limited. This research aims to examine the relationship between film thickness, performance, and aging behavior of HCPMA mixtures in order to establish an optimal film thickness that ensures satisfactory performance and aging durability. HCPMA specimens with film thicknesses ranging from 6.9 μm to 17 μm were prepared using a 7.5% SBS-content-modified bitumen. Various tests, including Cantabro, SCB, SCB fatigue, and Hamburg wheel-tracking tests, were conducted to evaluate raveling, cracking, fatigue, and rutting resistance before and after aging. The key findings indicate that insufficient film thickness negatively affects aggregate bonding and performance, while excessive thickness reduces mixture stiffness and resistance to cracking and fatigue. A parabolic relationship between the aging index and film thickness was observed, suggesting that increasing film thickness improves aging durability up to a point, beyond which excessive thickness adversely impacts aging durability. The optimal film thickness for HCPMA mixtures, considering performance before and after aging and aging durability, falls within the 12.9 to 14.9 µm range. This range ensures the best balance between performance and aging durability, offering valuable insights for the pavement industry in designing and utilizing HCPMA mixtures.

## 1. Introduction

Open-graded friction courses (OGFCs) have been utilized since the 1950s in Europe and the United States to enhance the frictional resistance of asphalt pavements [1,2,3]. Early research on porous asphalt pavement in China began in the 1980s [4], while Japan imported its initial porous asphalt experience from Europe in 1987. In Malaysia, early applications of porous asphalt occurred in the 1990s on JKR roads [5]. The Chinese government has recently promoted the ‘Sponge City’ strategy to encourage widespread adoption of OGFCs [6]. However, porous asphalt mixtures, such as OGFCs, pose challenges to the performance of asphalt binders due to their open-graded structure, which makes them more susceptible to environmental and traffic-related stresses. Conventional SBS-modified asphalt, containing 3% to 4.5% SBS content, performs adequately under certain conditions but may face limitations when it comes to resisting shear stress and heavy loads in specific applications, such as OGFCs. To address these performance demands, a novel asphalt binder known as high-content polymer-modified asphalt (HCPMA) was introduced in the early 2000s. HCPMA features an SBS concentration of 5% to 15%, offering improved properties over conventional SBS-modified asphalt, particularly when applied in OGFCs. The aging issues are especially pronounced in porous asphalt mixtures due to their open-graded structure, which results in the excessive oxidation of bitumen. HCPMA, with its high SBS content, exhibits certain challenges related to aging. The susceptibility of SBS polymers to aging can be attributed to the presence of unsaturated double-carbon bonds in its molecular structure in SBS polymers [7]. Over time, as the SBS polymer undergoes degradation, the polymer network within HCPMA may be compromised, resulting in a significant decline in the material’s rheological properties.

Numerous studies have been carried out on HCPMA; however, a comprehensive understanding of its modification mechanism and aging properties remains elusive. Several researchers have examined the effects of incorporating a plasticizer (furfural extract oil) and a cross-linker (sulfur) on the rheological properties of HCPMA [8,9,10]. Nevertheless, the SBS content in their studies was only 6%, significantly lower than the concentration employed in real-world applications. In another investigation, Zhang [11] delved into the composition and aging of HCPMA asphalt, proposing that increasing the SBS polymer content serves as one of the most effective methods for maintaining its physical properties. Furthermore, Jing and Vaveri explored the aging properties of bitumen’s microscale aging behavior, examining samples from both laboratory and field settings using chemical and rheological tests [12]. Wu and Hou, on the other hand, employed Fourier transform infrared (FTIR) spectroscopy and atomic force microscopy (AFM) techniques to analyze changes in the morphology and chemical structure of SBS-modified binders [13,14].

Despite the durability concerns surrounding HCPMA, limited investigations have been conducted on its aging behavior at the mixture level. Ruan et al. concluded that oxidative aging damages the polymer network in SBS-modified bitumen, resulting in decreased temperature susceptibility, a broader relaxation spectrum, and reduced polymer modification effectiveness for enhancing asphalt mixture ductility [15]. Cuciniello et al. suggested that SBS polymer degradation can counteract the stiffening of the bitumen phase in HCPMA during oxidative aging [16]. SBS polymer degradation plays a dominant role, particularly when the SBS content by weight of the total binder exceeds 6%, leading to increased nonrecoverable compliance. Mackiewicz et al. discovered that asphalt mixtures with a 7% polymer-modified binder were most resistant to permanent deformations [17]. Lin et al. noted that due to HCPMA’s complex aging behavior, an overall evaluation of resistance to permanent deformation should be determined for asphalt mixtures after both short- and long-term aging [7]. Yan et al. examined the impact of HCPMA aging using the Cantabro loss test, semicircular bend (SCB) test, and Hamburg wheel-tracking (HWT) test [18]. They observed an inconsistency between the low nonrecoverable compliance value (Jnr3.2) of an extracted binder and the deep, rutting depth of the asphalt mixture after 2 h of aging at 210 °C, indicating that the Jnr3.2 from the MSCR test might overestimate HCPMA’s rutting resistance [19].

The bitumen film thickness in asphalt mixtures significantly influences aging, permeability, and stability, prompting researchers to propose it as a criterion for ensuring the durability of asphalt mixtures. Campen et al. [20], Goode et al. [21], and Kumar and Goetz [22] studied the tensile strength and resilient modulus of asphalt mixtures with varying effective film thicknesses and aging degrees. They also analyzed the penetration, viscosity, complex modulus, and phase angle of recovered binders from these mixtures, concluding that film thickness significantly affects the aging hardening of bitumen and asphalt mixtures. Kandhal and Chakraborty [23] investigated the impact of bitumen film thickness on the short-term and long-term aging of asphalt mixtures to establish a relationship between film thickness and aging characteristics. They determined that aging accelerated when the bitumen thickness was less than 9 μm and proposed that a minimum average film thickness of 8 μm is more rational than specifying a minimum void in the mineral aggregate (VMA), as recommended by McLeod and adopted by Superpave [24,25].

Sengoz and Topal [26] all found that an optimum bitumen film thickness of about 9–10 μm is necessary to prevent accelerated aging and ensure adequate resilient modulus and indirect tensile-strength values in hot-mix asphalt, with a minimum VMA of 15.2% required to achieve this thickness. Dong et al. [27] observed that the asphalt film failure mode changes from adhesion to cohesion failure when the bitumen film thickness increases from less than 23.61 μm to over 219 μm. Kandhal and Chakraborty [28], Sengoza and Agarb [29], Yang and Jiang [30]. Zhang [31,32,33] reported that the bitumen film thickness also has significant influence on the aging effect and fatigue performance. In summary, the bitumen film thickness in asphalt mixtures plays a crucial role in determining pavement durability and performance, with the optimum thickness depending on various factors, such as binder type, asphalt mixture, aggregate gradation, and environmental conditions.

As it is challenging to measure the actual bitumen film in asphalt mixtures, the bitumen film thickness is a conceptual average value, with different methods developed for its calculation. Initially, calculations were based on an aggregate surface-area determination, which assumed that the effective bitumen binder coated aggregate particles with an equal film thickness [20]. However, this method did not consider air voids or voids of mineral aggregate (VMA). Boris Radovskiy proposed a new definition of film thickness, as well as analytical formulas for calculating the film thickness considering VMA [34]. Heitzman [35] developed a method to include the particle-shape effect of the aggregate in bitumen film-thickness calculations and conducted a sensitivity analysis of various factors for different methods. Despite the limitations of the aggregate shape investigation, the most widely used bitumen film-thickness calculation remains based on aggregate surface area factors, as described in the Asphalt Institute Manual, Series No. 2 [36].

## 2. Objective

Previous research has demonstrated the critical role of asphalt mixture film thickness in determining performance and aging behavior, and optimum film thicknesses have been established for various mixtures. However, the application of HCPMA in porous asphalt is relatively new, and the appropriate film thickness and its influence on performance and aging behavior remain unclear. Durability is a vital factor in determining the performance of HCPMA porous asphalt, and ensuring an adequate bitumen film thickness is essential for acceptable durability. Current pavement specifications (EN 13108-1:2016) have minimum bitumen content requirements to ensure durability, which may not be suitable for HCPMA porous asphalt. This study aims to identify the optimum bitumen film thickness for HCPMA porous asphalt, ensuring satisfactory durability based on aging characteristics. The primary objectives of this investigation are:

(1) To evaluate the impact of bitumen film thickness on HCPMA porous asphalt mixture performance by assessing its indirect tensile strength, rutting resistance, fatigue resistance, and raveling resistance.
(2) To examine the aging behavior of an HCPMA porous asphalt mixture and establish the correlation between bitumen film thickness and its aging durability.
(3) To recommend an optimum bitumen film thickness for an HCPMA porous asphalt mixture that ensures satisfactory performance and aging durability, based on the characterization of its performance and aging behavior.

## 3. Materials and Methods

### 3.1. Bitumen and Aggregate

In this study, a high-content polymer-modified asphalt (HCPMA) containing 7.5% linear SBS polymer (30% styrene) was used as a representative sample. The HCPMA was prepared in the laboratory based on the literature and previous research [3,7], using a shearing machine and a blending machine through the following process: First, the SBS polymer was added to the base bitumen at 185 °C and sheared at 4000 rpm for 30 min. Next, the sheared binder was mixed using a mechanical blender at 500 rpm for an hour. Finally, 0.15 wt% sulfur was added to enhance the storage stability, and the resulting mixture was blended for an additional 30 min. The conventional and rheological properties of the HCPMA are presented in Table 1.

In order to evaluate the influence of bitumen film thickness on the aging characteristics of asphalt mixture, the basalt aggregates and limestone filler were used to prepare the mixture specimens. The aggregate properties of the basalt aggregate and limestone filler can be seen in Table 2.

### 3.2. Mixture Design and Preparation

The typical open-graded friction course with 13 mm nominal maximum aggregate sizes (OGFC-13) was investigated, with three distinct gradations—fine, mid, and coarse—being selected to represent the different particle size distributions within the HCPMA mixtures. Additionally, three bitumen content values (4%, 5%, and 6%) were chosen for preparing the HCPMA-mixture specimens, maintaining the air void at 20%. Detailed gradations can be found in Table 3. In drainage-wearing courses, where HCPMA is employed as the binder, fibers are not commonly used in porous asphalt mixtures due to the high strength and viscosity of HCPMA. Furthermore, the primary objective of this research is to examine the influence of film thickness on the performance of porous asphalt. Consequently, no fibers were incorporated into the mixtures in this study.

The mixture specimens were prepared using the superpave gyratory compactor (SGC) at 185 °C, following field-experience guidelines. Film thickness, a crucial factor affecting the performance and durability of HCPMA mixtures, was calculated using the Asphalt Institute Manual, Series No. 2 (MS-2) [36]. This method relies on the surface-area (SA) factors for each sieve size specified in the aggregate gradation. The average film thickness can be determined using the following equation:

(1) $$T_{F}=1000\times \frac{P_{be}}{SA\times P_{s}\times G_{b}}$$

where:

*T_F_* = average film thickness (μm);
*P_be_* = percentage (by weight) of effective asphalt binder in the mix;
*SA* = surface area of aggregate gradation (m^2^/kg);
*P_s_* = percentage (by weight) of aggregate;
*G_b_* = specific gravity of asphalt binder.

According to the Asphalt Institute MS-2, the surface area of the aggregates can be calculated from gradation based on the percentage, passing a set of sieves as follows:

(2) $$SA=0.01\sum_{i}^{N}{PP}_{i}\times {CP}_{i}$$

where:

*N* = number of sieves considered in the surface area calculation;
*PP_i_* = percentage of aggregates passing sieve *i* (defined for sieves of 9.5, 4.75, 2.36, 1.18, 0.60, 0.30, 0.15, and 0.075 mm);
*CP_i_* = surface area factor outlined in Asphalt Institute MS-2.

Utilizing Equations (1) and (2), the bitumen film thickness can be determined, and the corresponding results are presented in Table 3. To categorize the HCPMA mixtures, they were labeled based on their approximate bitumen film thickness. For instance, HCPMA-12.9 μm refers to an asphalt mixture with an approximate film thickness of 12.9 μm.

### 3.3. Aging Process of Asphalt Mixture 

**Figure 1:**
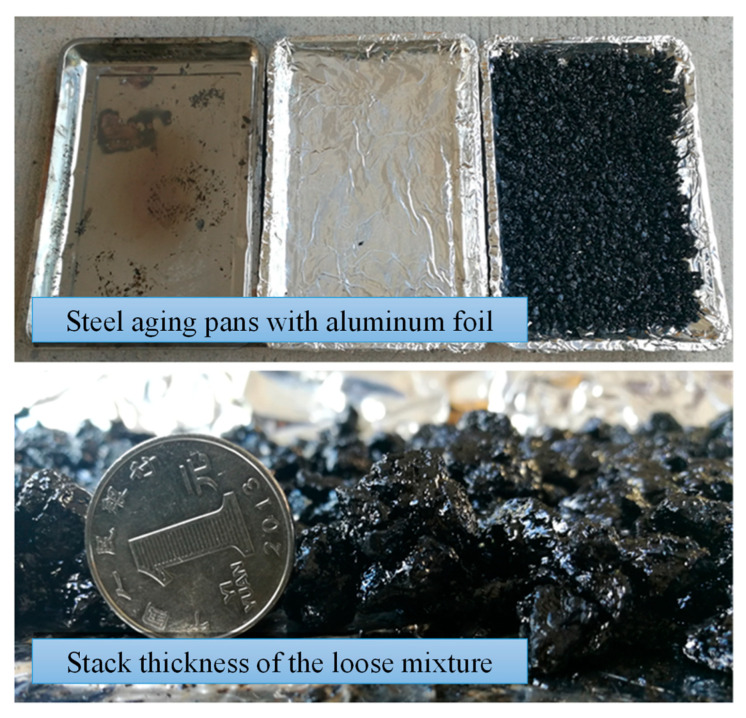
Schematic diagram of aging pan and loose HCPMA mixture.

In this study, both short-term and long-term aging procedures were conducted in accordance with AASHTO R30. The blended loose mixture was placed on a steel pan lined with aluminum foil to prevent the asphalt mixture from adhering to the pan. The thickness of the asphalt mixture was maintained below 1 inch (25.4 mm) to ensure consistent and uniform aging (Figure 1). The standard short-term aging temperature of 135 °C was deemed unsuitable for this study, as it is significantly lower than the mixing and transport temperature of HCPMA and does not accurately represent field conditions. Consequently, short-term aging was carried out at the compaction temperature of HCPMA (163 °C) for 2 h to simulate the aging process during the mixing, transportation, and paving stages [19]. The mixture was stirred every 60 min to ensure uniform aging throughout the process.

Long-term aging was also performed in compliance with AASHTO R30 to emulate the in-field aging of HCPMA. Following the short-term aging, the loose HCPMA was compacted using a superpave gyratory compactor (SGC). Once cooled to an ambient temperature, the compacted HCPMA specimens were placed in an oven set at 85 °C for 120 h to undergo long-term aging. Subsequently, the HCPMA specimens were allowed to cool to environmental temperature for 16 h, after which the long-term aged specimens were prepared for subsequent testing.

### 3.4. Test Methods

#### 3.4.1. Cantabro Loss Test

In order to evaluate the raveling resistance of the porous asphalt mixture, the Cantabro test was applied in this research. Following the standard of AASHTO TP 108-14, a cylindrical asphalt mixture specimen with a 100 mm diameter and a 63.5 mm height was cured in a 25 °C bath for 20 h. Then the mixture specimen was placed into the Los Angeles abrasion machine, and the machine was rotated for 300 revolutions at a speed of 30 rpm at 25 °C. The percentage of the mass loss was calculated to represent the raveling of the asphalt mixture. For each type of mixture, four replicates were conducted.

#### 3.4.2. Semicircular Bending (SCB) Strength Test

The cracking resistance of HCPMA was evaluated using the semicircular bend (SCB) strength test. In accordance with previous research and preparation methodologies [37], cylindrical specimens measuring 135 mm in height and 150 mm in diameter were prepared using a superpave gyratory compactor. Subsequently, each cylindrical specimen was cut into four semicircular specimens with dimensions of 50 mm in height and 150 mm in diameter. A notch, 15 mm in length and 1.5 mm in width, was cut at the center bottom of each specimen to ensure the appropriate cracking mode. The mixture specimens were preconditioned in a test chamber at 25 °C for a minimum of 4 h before conducting the SCB strength test at a displacement rate of 50 mm/min and a temperature of 25 °C. Based on the force-displacement curve, tensile strength ($\sigma_{max}$) and fracture energy ($G_{f}$) were calculated using the following equations.

Tensile strength ($\sigma_{max}$) represents the asphalt mixture’s strength and is calculated using the equation:

(3) $$\sigma_{max}=\frac{4.263\times F_{max}}{D\times t}$$

where *F_max_* is the maximum force in N, *D* is the specimen’s diameter in mm, and *t* is the specimen’s thickness in mm. The constant 4.263 is derived from research by Van de Ven et al. by using 3D finite element analysis, assuming a support span of 80% of the specimen’s diameter [37].

Fracture energy ($G_{f}$) represents the amount of energy consumed to create cracks per unit area and is calculated using the equation:

(4) $$G_{f}=\frac{W_{f}}{A_{lig}}$$

where ($W_{f}$) (fracture work) is the work done during the fracture process, calculated as the area under the force-displacement curve. $A_{lig}$ is the ligament area, calculated as:

(5) $$A_{lig}=\left(W-a\right)\times T$$

where *W* is the specimen’s height in mm, *a* is the depth of the specimen’s notch in mm, and *T* is the specimen’s thickness in mm.

#### 3.4.3. Semicircular Bending (SCB) Fatigue Test

The fatigue resistance of HCPMA was assessed using the SCB fatigue test. As documented in the literature, the stress-controlled SCB fatigue test is a widely utilized method, and in this study, four stress amplitudes (stress ratio ($\sigma_{ratio}$) of 0.3, 0.4, 0.5, and 0.6) were chosen and applied in the SCB fatigue tests [38,39,40]. In this research, the stress level is defined as the ratio of stress amplitude to the tensile strength of the specimen [39].

Prior to the SCB fatigue test, the SCB strength test was performed on four replicate specimens to determine their tensile strengths at 15 °C. Once the tensile strength values were obtained, stress amplitudes were calculated using the selected stress ratios (0.3, 0.4, 0.5, and 0.6) and then applied to the SCB fatigue test. The frequency of the repeated compressive load was set at 10 Hz, comprising a 0.1 s half-sine load and no rest period under different stress levels. Four replicate SCB fatigue tests were conducted at 15 °C for each HCPMA type at each stress level.

To further analyze the SCB fatigue results, a function can be utilized to describe the relationship between the fatigue life and the stress ratio. The literature shows a linear relationship between stress ratio and fatigue life in double logarithmic coordinates [39]. Thus, the fatigue life can be described with Equations (6) and (7). The expression between $lg(N_{f})$ and $lg(\sigma_{ratio})$ is a simple linear relationship. Based on the SCB fatigue results, the least-squares equation can be obtained, and then the parameters *a* and *b* are determined correspondingly.

(6) $$N_{f}=a{\left(\sigma_{ratio}\right)}^{b}$$

(7) $$lg(N_{f})=lga+b\times lg(\sigma_{ratio})$$

where

$N_{f}$ is the fatigue life in the SCB fatigue test;
$\sigma_{ratio}$ is the stress ratio, which is the ratio between the loading stress in the SCB fatigue test and the peak stress in the SCB strength test;
*a* is a regression parameter that describes the fatigue life of the specimen;
*b* is a regression parameter that describes the stress sensitivity of the specimen.

#### 3.4.4. Hamburg Wheel-Tracking (HWT) Test

The rutting resistance of the HCPMA mixture was evaluated using the Hamburg wheel-tracking (HWT) test. The HWT test was performed with a double wheel track (DWT) device from the Controls Group, Milan, Italy, at 60 °C under moist conditions, in accordance with AASHTO T324-11. Two cylindrical specimens, each measuring 150 mm in height and 62 mm in diameter, were loaded into the HWT device as a single test sample. The HCPMA mixture specimens were subjected to steel wheels rolling at a speed of 52 passes/min, while a linear variable differential transformer (LVDT) was employed to record the specimens’ relative vertical deformation. The test concluded either when the vertical deformation reached 20 mm or the specimen had undergone 20,000 load passes.

Following the HWT test, a rutting depth-load number curve was generated for each test sample, with the results representing the average of two test repetitions. Rutting resistance can be characterized by the slope of the creep stage, while stripping resistance can be characterized by the slope of the stripping stage. It should be noted that the stripping stage is considered only when the stripping stage slope is at least twice the creep slope. The consistency and reliability of the two replicates were observed, and any variations between the repetitions were reported in the manuscript.

#### 3.4.5. Aging Index of Performance Parameters 

To assess the impact of bitumen film thickness on the aging durability of HCPMA porous asphalt mixtures, it is crucial to establish a reliable and robust metric capable of effectively quantifying the aging process. For this purpose, a series of aging indices are proposed, which are based on the ratio of performance evaluation parameters before and after aging. These parameters include the Cantabro loss ratio, fracture strength, fracture energy, fatigue parameters, and creep slope. These aging indices offer a standardized measure of the degradation extent resulting from the aging process, enabling a comprehensive comparison of the performance of different mixtures with varying bitumen film thicknesses. The aging index can be defined as follows:

(8) $$Aging\;Index\;\left(AI\right)=\frac{{Performance\;Parameter}_{afteraging}}{{Performance\;Parameter}_{beforeaging}}\times 100\%$$

The significance of these aging indices lies in their ability to quantify the impact of aging on the HCPMA porous asphalt mixtures and correlate this degradation with the bitumen film thickness. The aging indices will provide a valuable tool for pavement engineers and researchers to assess and monitor the performance of HCPMA porous asphalt mixtures under different aging conditions, ultimately contributing to the design and construction of more durable and sustainable asphalt pavements.

## 4. Results and Discussion

### 4.1. Cantabro Loss Test Results

**Figure 2:**
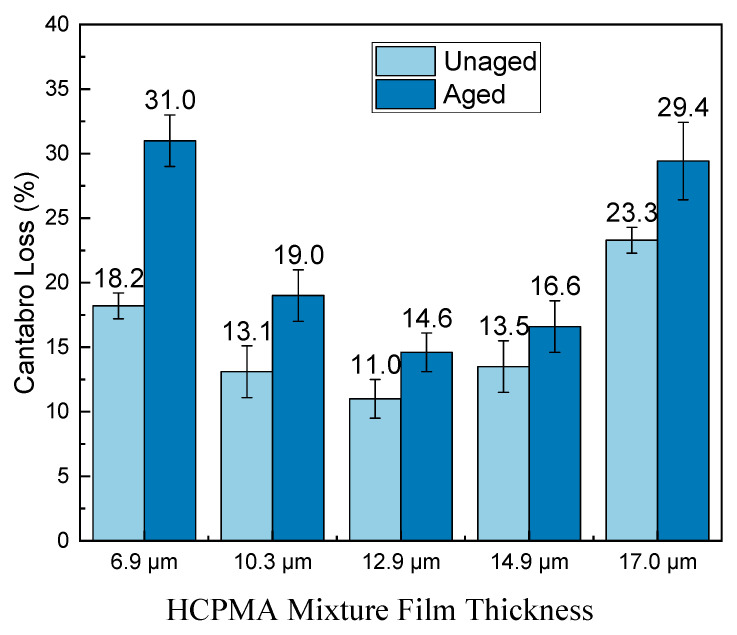
Influence of bitumen film thickness on the raveling resistance of HCPMA.

The Cantabro test is designed to evaluate the adhesive and cohesive properties of high-content polymer-modified asphalt (HCPMA) and assess its resistance to raveling. The results of HCPMA before and after aging can be seen in Figure 2.

In the unaged HCPMA analysis, a noticeable relationship between film thickness and performance was observed in the Cantabro loss results. As film thickness increased from 6.89 µm to 12.9 µm, Cantabro loss declined from 18.2% to 11.0%. However, upon further increasing the film thickness to 17.0 µm, the Cantabro loss rose to 23.3%. This suggests that 12.9 µm is the optimal film thickness for achieving the best adhesive, cohesive, and raveling-resistance properties. When compared to the CROW guidelines (Netherlands Centre for Research on Road Engineering) standard, which stipulates a maximum Cantabro loss of 20%, HCPMA mixture samples with film thicknesses of 14.9 µm or less met the requirement. This acceptable performance can likely be attributed to the high styrene–butadiene–styrene (SBS) content in HCPMA. The analysis of unaged samples indicates that insufficient film thickness leads to ineffective aggregate bonding, while excessive film thickness results in reduced stiffness and compromised adhesive and cohesive performance. Consequently, a film thickness of 12.9 µm is deemed optimal.

For aged HCPMA samples, the film thickness’s impact on Cantabro loss is akin to that of the unaged samples. The Cantabro loss decreased from 31.0% to 14.6% when the film thickness increased from 6.89 µm to 12.9 µm. However, further increasing the film thickness to 17.0 µm resulted in a Cantabro loss of 29.4%. Thus, the optimal film thickness of 12.9 µm for adhesive, cohesive, and raveling-resistance properties remains consistent after aging. In comparison to the CROW-2015 standard, HCPMA samples with film thicknesses of 6.89 µm and 17 µm did not satisfy the 20% Cantabro loss requirement after aging, possibly due to HCPMA aging and the reduced effectiveness of its high-SBS content. After aging, the influence of film thickness on adhesive performance remained in line with the observations from unaged samples, with 12.9 µm remaining as the optimal thickness.

**Figure 3:**
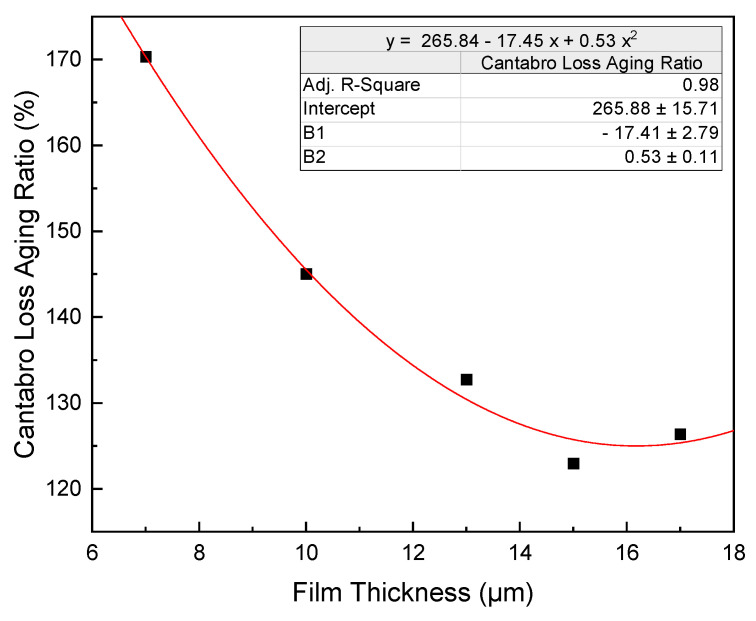
Influence of bitumen film thickness on the aging ratio of Cantabro loss.

The aging index of Cantabro loss is defined as the ratio between the Cantabro loss after aging and the Cantabro loss before aging. The results can be seen in Figure 3. Lower Cantabro loss values indicate a better raveling resistance, while higher aging index values suggest reduced aging durability. As observed in Figure 3, the aging index decreases considerably as the film thickness increases, following a parabolic relationship with film thickness.

In summary, increasing film thickness enhances aging durability up to a point, but beyond that point, it offers diminishing returns. The optimal film thickness before and after aging for adhesive and cohesive performance is 12.9 µm. Film thickness beyond 12.9 µm provides limited improvements in aging durability.

### 4.2. SCB Cracking Test

**Figure 4:**
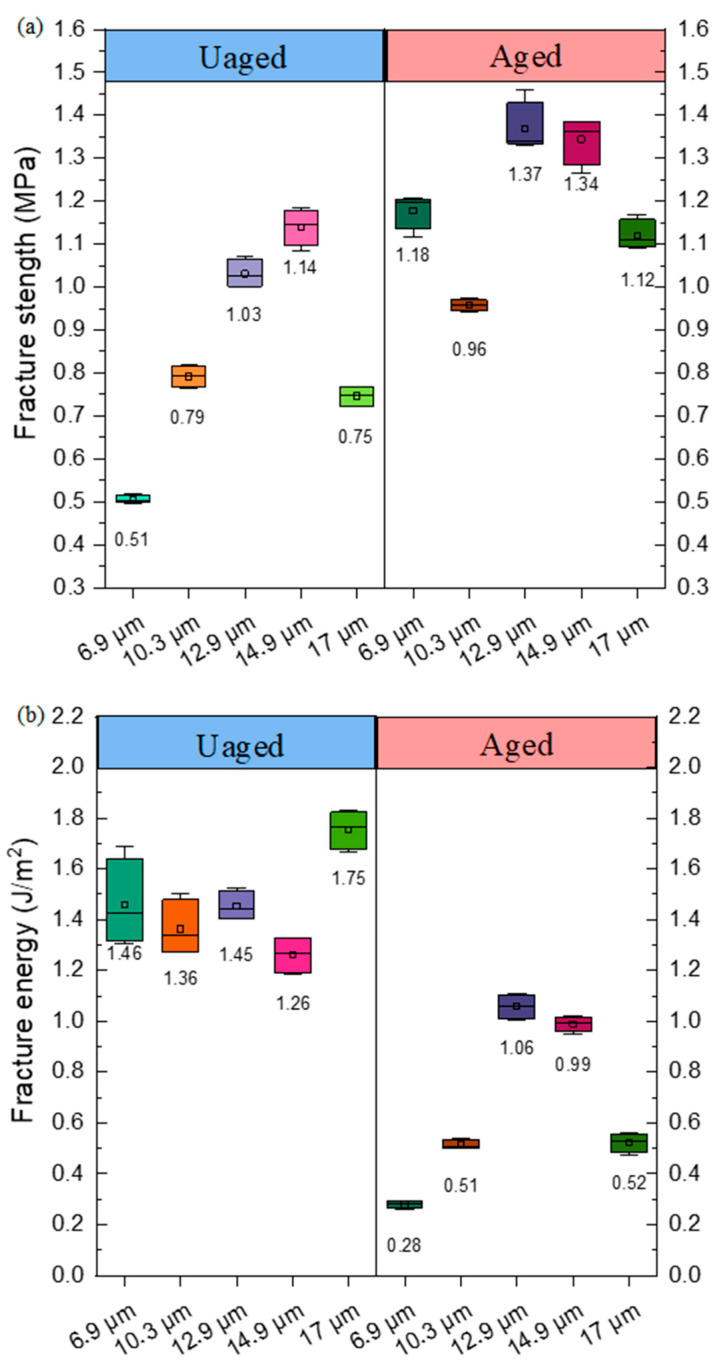
Influence of bitumen film thickness on SCB results of HCPMA: (**a**) fracture strength and (**b**) fracture energy.

The semicircular bend (SCB) test aims to characterize the cracking resistance properties of HCPMA and the influence of aging on its cracking resistance. The SCB cracking results of HCPMA before and after aging can be seen in Figure 4a,b.

Regarding the fracture strength, for unaged samples, the strength increased from 0.51 MPa to 1.14 MPa when the film thickness increased from 6.9 µm to 14.9 µm, and the fracture strength decreased to 0.75 MPa when the film thickness reached 17 µm. The optimal film thickness considering the fracture strength of HCPMA before aging was 14.9 µm. For aged samples, the fracture strength increased significantly during aging, likely due to bitumen oxidation, which increased the stiffness of HCPMA. The fracture strength increased from 1.18 MPa to 1.37 MPa when the film thickness increased from 6.9 µm to 12.9 µm and decreased to 1.12 MPa when the film thickness reached 17 µm. The optimal film thickness, considering the fracture strength of HCPMA after aging, was 14.9 µm.

As for the fracture energy, before aging, the influence of film thickness on fracture energy was not significant, with values ranging from 1.26 J/m^2^ to 1.75 J/m^2^ when the film thickness increased from 6.9 to 17 µm. There was no clear trend between film thickness and fracture energy. After aging, the fracture energy decreased significantly due to bitumen oxidation and SBS polymer degradation in HCPMA, leading to a decreased ability to absorb energy during cracking. The fracture strength after aging increased from 0.28 MPa to 1.06 MPa when the film thickness increased from 6.9 µm to 12.9 µm and decreased to 0.52 MPa when the film thickness reached 17 µm. The optimal film thickness, considering the fracture strength of HCPMA after aging, was 12.9 µm.

**Figure 5:**
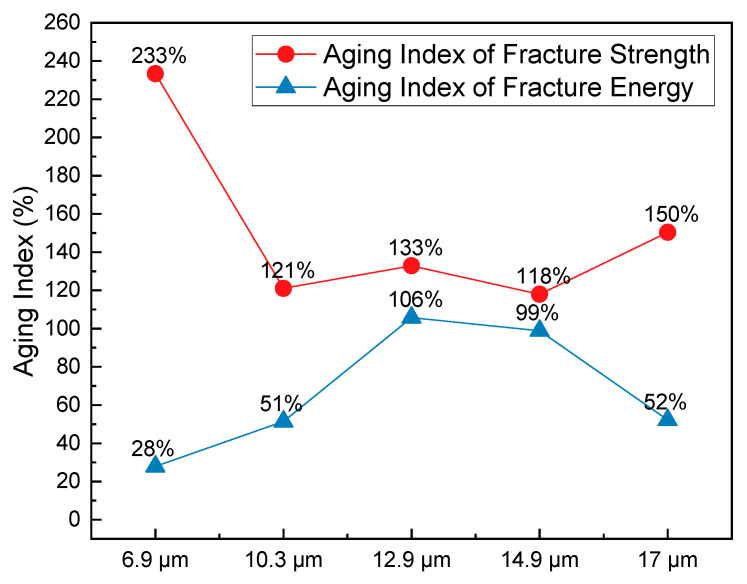
Influence of bitumen film thickness on the fracture strength of HCPMA.

The aging indices of fracture strength and fracture energy provide a quantitative assessment of the change in cracking resistance properties of HCPMA before and after aging, and the results can be seen in Figure 5. By comparing these indices, we can evaluate the impact of aging on the performance of HCPMA mixtures and the influence of film thickness on the aging durability.

For the fracture strength, the aging index decreased from 233% to 118% when the film thickness increased from 6.9 µm to 14.9 µm. However, when the film thickness reached 17 µm, the aging index of fracture strength increased to 150%. This indicates that an increase in film thickness up to 14.9 µm improves the aging durability in terms of fracture strength, but beyond that point, the benefits are limited. On the other hand, the aging index of fracture energy showed an inverse relationship with film thickness. The index increased from 28% to 106% when the film thickness increased from 6.9 µm to 12.9 µm and decreased to 52% when the film thickness was 17 µm. This suggests that the aging durability, in terms of fracture energy, is more sensitive to changes in the film thickness and is best at 12.9 µm.

While the aging indices of fracture strength and energy show contrary responses to the increase in film thickness, it is important to note that fracture energy is a more critical parameter for cracking resistance. This is because cracking resistance is not determined solely by the strength and stiffness of the mixture but also by the ductility and ability to absorb energy during cracking. Considering both the fracture strength and fracture energy aging indices, a film thickness of 12.9 µm provides the best balance for aging durability and cracking resistance in HCPMA mixtures.

In light of the SCB test results and discussions, the optimum film thickness for the HCPMA mixture’s cracking resistance and aging durability has been identified. Considering both unaged and aged HCPMA mixtures, the performance perspective suggests that the optimum film thickness for fracture strength and fracture energy lies within the range of 12.9 to 14.9 µm. Meanwhile, from an aging durability perspective, a 12.9 µm film thickness is found to be ideal for maintaining the cracking resistance of the HCPMA mixture as it demonstrates a better balance between the aging indices of fracture strength and fracture energy, which are essential indicators of the mixture’s long-term performance.

### 4.3. SCB Fatigue Test Results

**Figure 6:**
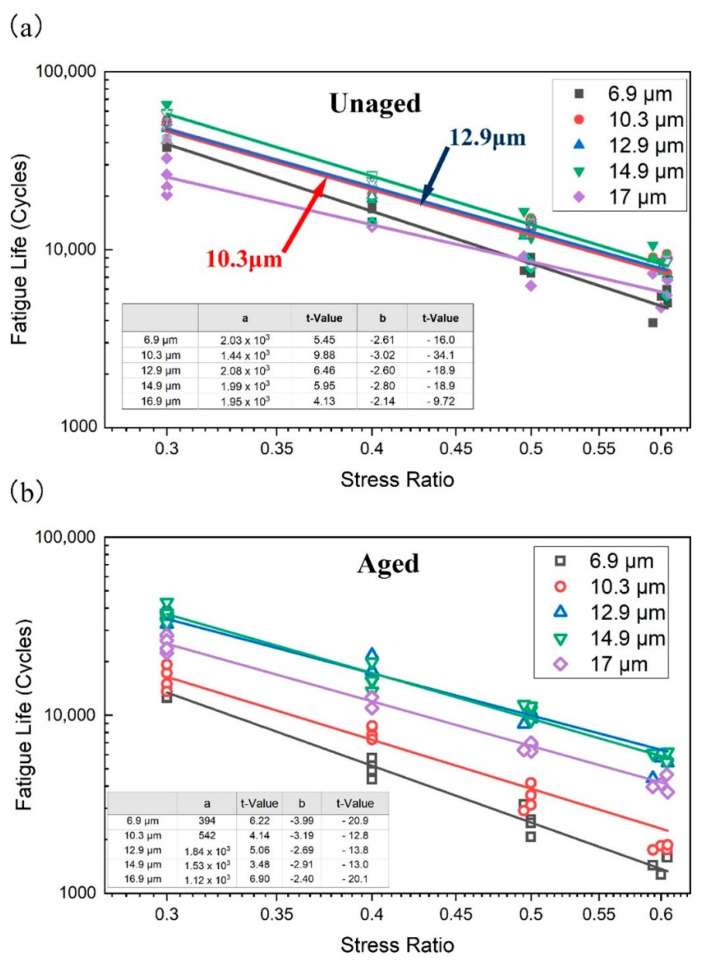
Influence of bitumen film thickness on the fatigue life at different stress ratios, (**a**) fatigue life before aging, (**b**) fatigue life after aging.

The SCB fatigue test is designed to characterize the fatigue performance of HCPMA, as well as to analyze the influence of aging on its fatigue performance. This can be observed through the SCB performance results of HCPMA before and after aging, as depicted in Figure 6a,b.

Before aging, the fatigue life of HCPMA increased with the increase in film thickness, peaking at a film thickness of 14.9 µm. Beyond this point, the fatigue life decreased significantly. Furthermore, it was observed that the slope of the fatigue life decreased as the film thickness increased, indicating that the fatigue performance of asphalt mixture is less sensitive to the increase in the stress ratio. In contrast, after aging, the fatigue life of HCPMA decreased significantly, likely due to the oxidation of the bitumen and degradation of the SBS polymer, which reduces the flexibility of the HCPMA. When comparing HCPMA with different thicknesses after aging, it was observed that HCPMA, with a thickness of 6.9 µm, had the lowest fatigue life, and as the film thickness increased, the fatigue life also increased significantly. The fatigue life is no longer as sensitive to the increase in the stress ratio, reaching a maximum when the film thickness is 12.9 µm or 14.9 µm, and then decreasing when the film thickness reaches 17.0 µm.

The SCB fatigue results highlight that, when the film thickness is too thin, the HCPMA cannot provide sufficient coating, leading to poor bonding between the aggregates and the binder. This results in a weaker mixture that is more susceptible to cracking and fatigue failure under repeated loading. On the other hand, when the film thickness is too high, the excess binder can negatively impact the mechanical properties of the mixture, decreasing its stiffness and making it more susceptible to deformation under repeated loading, ultimately reducing its fatigue life.

**Figure 7:**
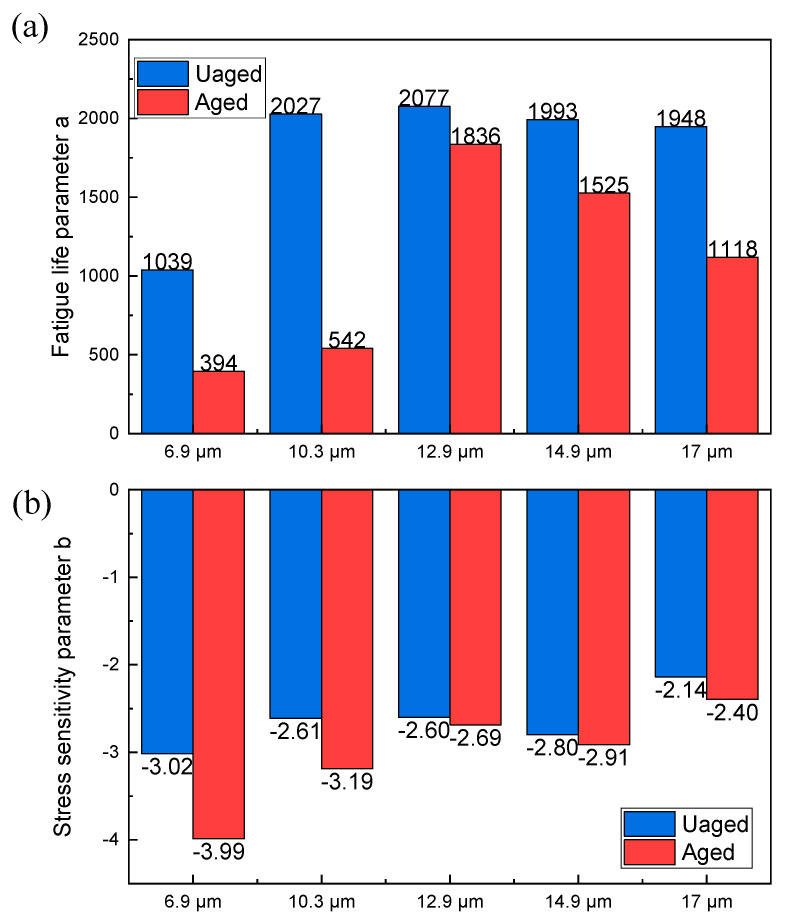
Influence of bitumen film thickness on the fatigue parameters of HCPMA, (**a**) influence on fatigue life parameter a, (**b**) influence on stress sensitivity b.

Fatigue parameters are essential for quantitatively characterizing the fatigue life of specimens, especially in the context of the SCB test results, which can be seen in Figure 7. In this analysis, the fatigue life is calculated using Equations (6) and (7). Two regression parameters are considered: fatigue life parameter a, which describes the fatigue life of the specimen, and stress sensitivity parameter b, which represents the stress sensitivity of the specimen.

Before aging, as the film thickness increased, fatigue life parameter a rose from 1039 to reach its highest value of 2077 at a film thickness of 12.9 µm. However, when the film thickness was further increased, this parameter decreased slightly to 1948 at 17 µm. After aging, fatigue life parameter a declined significantly, particularly for HCPMA with film thicknesses of 6.9 µm and 10.3 µm. This behavior suggests that, after aging, the fatigue parameter a becomes more sensitive to changes in film thickness. The high content of SBS in HCPMA contributes to the material’s excellent performance before aging, increasing its tolerance to variations in film thickness. However, after aging, the oxidation of the bitumen phase and degradation of the SBS polymer rendered the HCPMA more sensitive to film thickness.

The stress sensitivity parameter b exhibited a different trend. Before aging, this parameter increased from −3.02 to −2.14 as the film thickness increased from 6.9 µm to 17.0 µm. This indicates that a more considerable film thickness significantly reduces the stress sensitivity of HCPMA during fatigue loading. After aging, the stress sensitivity parameter b decreased significantly, particularly for HCPMA with film thicknesses of 6.9 µm and 10.3 µm. Similar to the parameter a, b also declined with increased film thickness after aging, suggesting that a greater film thickness decreases the stress sensitivity of HCPMA even after aging.

**Figure 8:**
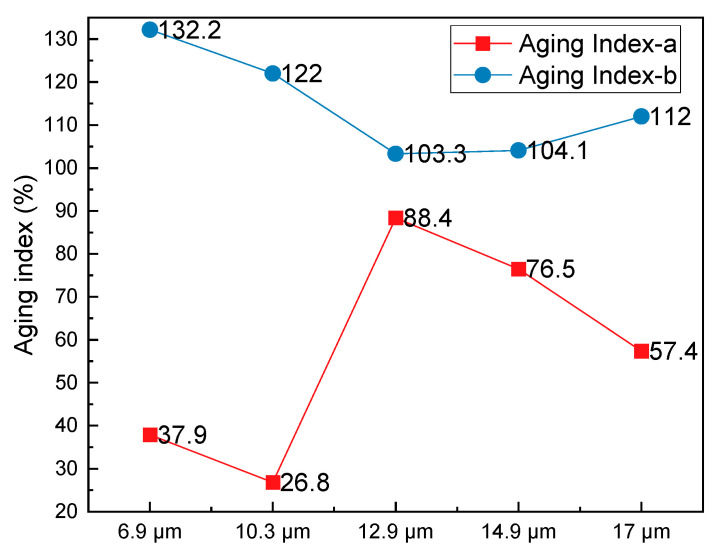
Influence of bitumen film thickness on the aging indices of fatigue parameters.

The aging index plays a crucial role in understanding the fatigue behavior of HCPMA specimens after undergoing the aging process. Defined as the ratio between fatigue parameters before and after aging, the aging index results can be observed in Figure 8.

Upon examining Figure 8, it is evident that the aging index for parameter a (Aging Index-a) increased from 37.9% to 88.4% as the film thickness rose from 6.9 µm to 14.9 µm. However, when the film thickness reaches 17 µm, aging index a increased to 57.4%. In contrast, the aging index for parameter ‘b’ (aging index b) decreased from 132.2% to 103.3% as the film thickness increased from 6.9 µm to 12.9 µm, before increasing again to 112% when the film thickness reached 17 µm. This contrasting influence of film thickness on the aging indices of parameters ‘a’ and ‘b’ is noteworthy. A lower aging index a and a higher aging index b indicates that the HCPMA has improved fatigue resistance after aging. Taking both aging indices into consideration, a film thickness of 12.9 µm appears to be optimal for achieving the best aging durability.

In summary, the fatigue performance of HCPMA can be effectively quantified using fatigue life parameter a and stress sensitivity parameter b. The behavior of both parameters is influenced by the film thickness, with the fatigue life parameter a increasing and then decreasing with film thickness, reaching its peak value at 12.9 µm for both before and after aging. The stress sensitivity parameter b decreased with increasing film thickness before and after aging, suggesting that a more significant film thickness contributes to reduced stress sensitivity. By considering both aging index a and aging index b, it is determined that a film thickness of 12.9 µm is optimal for ensuring the highest aging durability.

### 4.4. HWT Test Results

**Figure 9:**
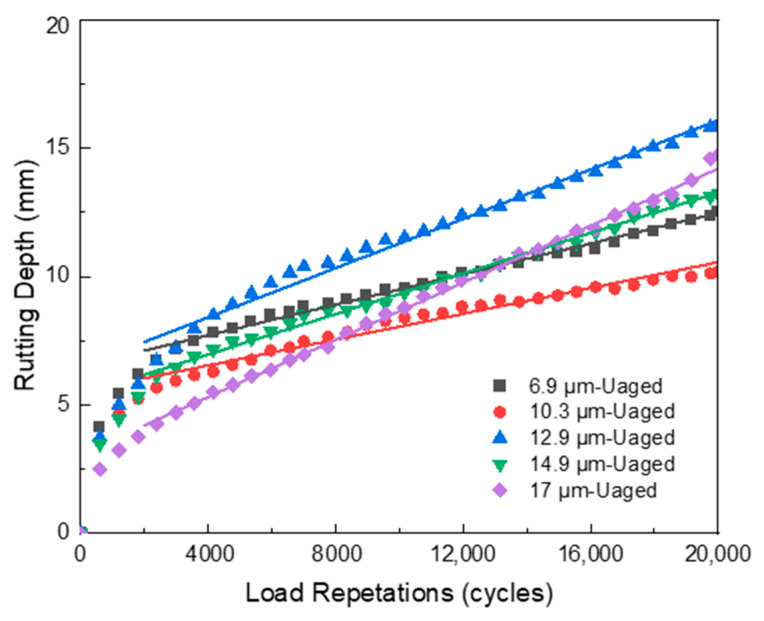
Influence of bitumen film thickness on the rutting resistance of HCPMA before aging.

**Figure 10:**
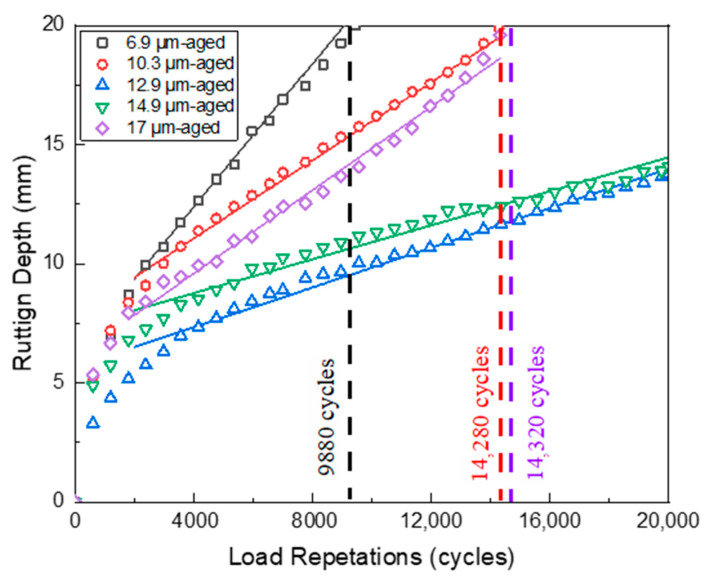
Influence of bitumen film thickness on the rutting resistance of HCPMA after aging.

The Hamburg wheel-tracking test (HWTT) was employed to assess the rutting and moisture susceptibility of HCPMA both before and after aging, by replicating real-world pavement conditions such as heavy traffic loads, repeated wheel passes, and water exposure. The results of the HWTT for HCPMA can be found in Figure 9 and Figure 10.

A typical rutting curve derived from the HWTT was divided into three distinct stages: postcompaction, creep, and stripping phases [41,42,43]. The post compaction phase involves the specimen’s consolidation as the wheel load compacts the mixture. During the creep phase, the deformation occurs primarily due to the viscous flow of the asphalt mixtures, which is characterized by a constant rate of increase in rut depth per load cycle (creep slope). The stripping phase commences when the bond between the asphalt binder and the aggregate weakens, leading to visible damage, such as stripping or raveling, as more load cycles are applied. The stripping inflection point (SIP) represents the number of load cycles at which a sudden increase in rut depth is observed, primarily as a result of asphalt binder stripping from the aggregate. The stripping stage is typically considered when the slope in the potential stripping region is twice the creep slope.

The HWTT results of the unaged HCPMA mixture, as depicted in Figure 9, demonstrate that the postcompaction phase occurs within the initial 2000 cycles. Subsequently, a clear and extended creep phase was observed, lasting for 20,000 cycles, until the conclusion of the test. A significant stripping phase is not present, as the slope does not increase to twice the creep slope, indicating that the HCPMA binder maintains a good bonding performance and resists the stripping of the binder from the aggregate. After 20,000 loading cycles, the rutting depths of all HCPMA samples remain under 20 mm, without a distinct relationship between rutting depth and film thickness. The highest rutting depth, measuring 16.5 mm, was attained when the HCPMA samples exhibited a film thickness of 12.9 µm.

The creep slope, which signifies the rate at which the asphalt mixture deforms under continuous loading, is of significant importance. The results reveal that, as film thickness increases, the creep slope also rises correspondingly. This relationship may be due to the vital role the aggregate skeleton plays in porous asphalt mixtures, offering effective load distribution, particle interlock, reduced reliance on binder, and increased stiffness, all of which contribute to enhanced rutting resistance. When film thickness increases, the mixture’s stiffness diminishes, which subsequently leads to an increased creep slope.

The aged HCPMA Hamburg wheel-tracking test (HWTT) results, as illustrated in Figure 10, provide insights into the performance and durability of the HCPMA porous asphalt mixtures after aging. The postcompaction phase of HCPMA samples after aging showed consistent loading cycles, ranging between 1 and 2000 cycles. This observation indicates that the aging process does not significantly alter the HCPMA mixtures’ response to the applied load cycles.

The HWTT results reveal a significantly steeper creep slope for the HCPMA samples after aging. With varying film thicknesses (6.9 µm, 10.3 µm, and 17 µm), the HCPMA mixtures reached a rutting depth of 20 mm after completing 9880, 14,280, and 14,320 load cycles, respectively. The steeper creep slope suggests a more rapid deformation of the asphalt mixture under sustained loading after aging, highlighting the impact of the aging process on the material’s performance. Furthermore, no significant stripping phase was observed after aging. This observation implies that the HCPMA binder maintains its excellent bonding properties, resisting the stripping of the binder from the aggregate even after the aging process. The absence of a stripping phase demonstrates the durability of the HCPMA binder in preserving adhesion to the aggregate throughout the aging process.

The analysis of the creep slope reveals a complex relationship between film thickness, performance, and aging durability. As the film thickness increased, the creep slope decreased considerably until a film thickness of 14.9 µm was reached. Beyond this point, the film thickness increased significantly when the thickness changed from 14.9 µm to 17 µm. When the film thickness is too low, the aging durability of the HCPMA cannot be guaranteed, resulting in a large creep slope after aging. Conversely, when the film thickness is too high (greater than 14.9 µm), the excess bitumen reduces the mixture’s stiffness, leading to a decrease in rutting resistance. These findings suggest that an optimal film thickness range between 12.9 and 14.9 µm allows the HCPMA to strike a balance between performance and aging durability, ensuring a reasonable creep slope, both before and after aging.

**Figure 11:**
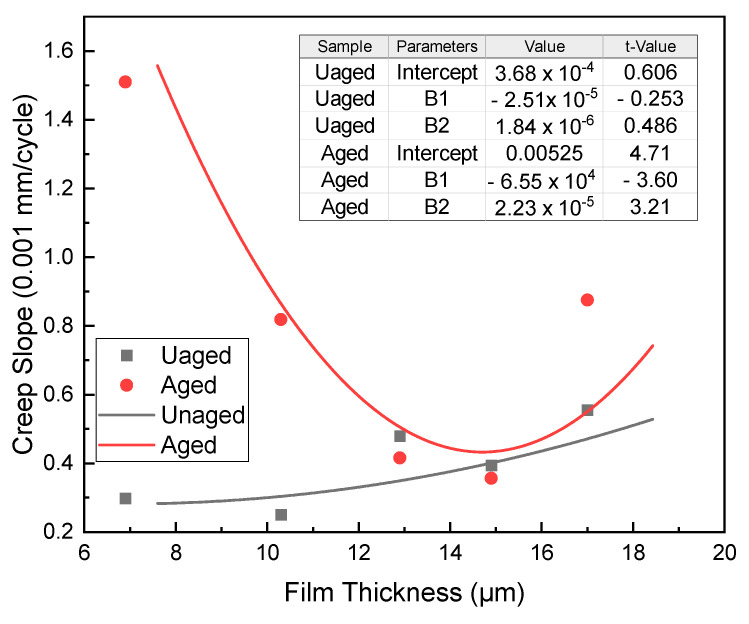
Influence of bitumen film thickness on the creep slopes before and after aging.

To quantitatively analyze the rutting curve, the data after 2000 cycles was fitted into a linear function, and the creep slope was obtained. This analysis provides a deeper understanding of the HCPMA’s performance in terms of rutting resistance, and the results can be seen in Figure 11.

Before aging, the creep slope remained at a very low level, indicating that the HCPMA demonstrates excellent rutting resistance. Additionally, the creep slope showed a slight increase with the rise in film thickness. After aging, the creep slope exhibited a significant increase, highlighting the effect of aging on the rutting resistance. The creep slope decreased from 1.52 to 0.42 (0.001 mm/cycle) when the film thickness grew from 7.9 to 14.9 µm. Conversely, the creep slope ascended from 0.42 to 0.92 (0.001 mm/cycle) when the film thickness expanded from 14.9 µm to 17 µm. A parabolic relationship was observed between the creep slope and film thickness, with the parameters of the fitting curve shown in Figure 11.

It is essential to note that the creep slope is not sensitive to changes in the film thickness before aging. However, after aging, the creep slope exhibited a parabolic relationship with film thickness, reaching its lowest point when the film thickness was approximately 14.9 µm. Taking into account the rutting resistance before and after aging, the optimal film thickness was determined to be 14.9 µm. This analysis provides valuable insights into the material’s performance and can inform future research and development efforts in the field of asphalt mixtures.

**Figure 12:**
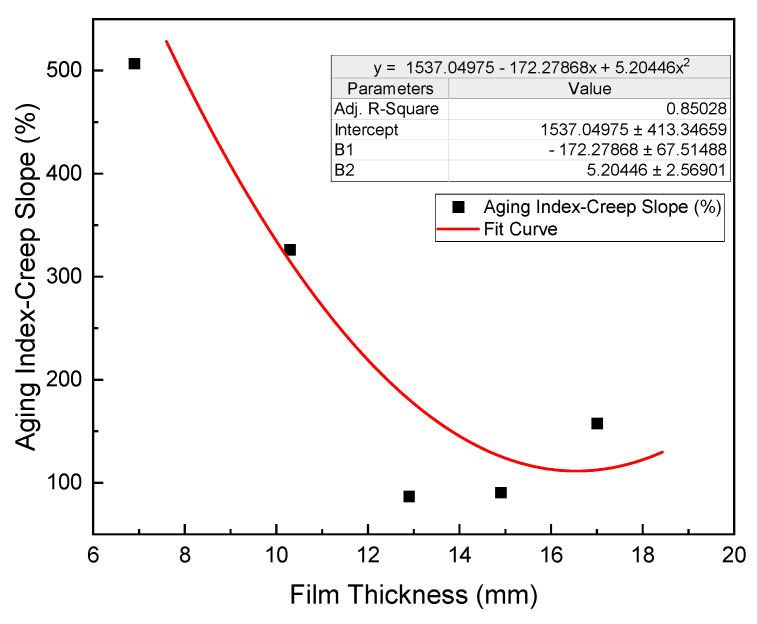
Influence of bitumen film thickness on Agin Index of creep slope.

Figure 12 displays the relationship between film thickness and creep slopes before and after aging, as well as the aging index for each respective film thickness. By examining Figure 12, it becomes apparent that the aging index of the creep slope, which is calculated as the ratio of creep slopes after aging to before aging, displays a nonlinear relationship with the film thickness.

To further explore this relationship, we first calculated the fitting function to model the parabolic relationship between the aging index and film thickness. The fitted function can be seen in Figure 12. The parabolic relationship between the aging index and the film thickness implies that there is a range of film thickness values that deliver good performances in terms of rutting resistance and aging durability. However, the performance deteriorates significantly for film thickness values outside of this range. As demonstrated in Figure 12, the optimal film thickness for HCPMA mixtures is approximately 14.9 µm.

In conclusion, our detailed analysis of the table and the derived fitting function indicates that the optimal film thickness for HCPMA mixtures is approximately 14.9 µm. This value provides a balance between rutting resistance and aging durability, ensuring satisfactory performance throughout the pavement’s service life. It is essential to maintain the film thickness within the optimal range to minimize the impact of the aging process on the performance of the asphalt mixture.

## 5. Conclusions and Recommendation

This study aimed to explore the relationship between film thickness and performance variations before and after aging and determine the optimal bitumen film thickness for high-content polymer-modified asphalt (HCPMA) mixtures to ensure satisfactory performance and aging durability by evaluating various properties. A range of experiments, including the Cantabro test, SCB test, SCB fatigue test, and Hamburg wheel-tracking test, were conducted, and the conclusions are as follows:

(1) Considering all of the tests, an insufficient film thickness was found to impair the bonding between aggregates and negatively affect performance, while an excessive film thickness reduced mixture stiffness, cracking resistance and fatigue performance. The optimal film thickness ranges between 12.9 µm and 14.9 µm for various properties, such as adhesive and cohesive performance, raveling resistance, fracture strength, and fatigue performance before and after aging.
(2) The experimental results obtained from the Cantabro test, SCB test, SCB fatigue test, and HWTT revealed a parabolic relationship between the aging index and film thickness, indicating that the increase in film thickness improved the aging durability, but a too-thick film thickness still harmed to the aging durability.
(3) The optimal film thickness, considering performance before and after aging and aging durability, is summarized in Table 4. Based on the results of all of the tests, the optimum film thickness for high-content polymer-modified asphalt (HCPMA) mixtures is within the range of 12.9 to 14.9 µm. This range ensures the best balance between performance before and after aging and aging durability.

Future research endeavors should address the limitations of this study so as to obtain a more comprehensive understanding of HCPMA porous asphalt mixtures. This would involve extending the investigation to encompass a wider variety of HCPMA materials, which would enhance the generalizability of the findings. Additionally, it would be beneficial to consider the impact of fiber reinforcement on the performance and aging behavior of the mixtures. Furthermore, a more in-depth analysis of failure modes during SCB tests could be conducted by using advanced imaging techniques, enabling the differentiation between adhesive and cohesive failures. By addressing these limitations and incorporating these future research directions, it will be possible to optimize the mixture designs, ultimately improving the performance and durability of HCPMA porous asphalt mixtures.

## Figures and Tables

**Table 1:** Conventional and rheological properties of HCPMA.

Parameters	HCPMA
SBS content	7.50%
Penetration at 25 °C, 0.1 mm	41
Softening point, °C	>100
Ductility at 5 °C, cm	44.1
135 °C, viscosity, Pa.s	5.72
70 °C complex modulus, Pa	5281
70 °C phase angle, °	48
70 °C Jnr3.2, kPa^−1^	0.011
70 °C R3.2, %	98.8
Elastic recovery at 25 °C, %	90.3
48 h softening-point difference, °C	1.8

**Table 2:** Basic properties of aggregate and filler.

Basic Properties	Basalt Aggregates/Limestone Filler
Flat and elongated particles of aggregate (%)	10.20
Fine aggregate angularity (%)	55.80
LA abrasion (%)	10.70
Fracture 1 face (%)	99.20
Fracture 2 face (%)	98.70
Water absorption of limestone filler (%)	1.21
Sand equivalent of fine aggregate (0.063–2.00 mm) (%)	72.00

**Table 3:** Asphalt mixture gradation and bitumen film thickness calculation.

Sieve Size	SA Factors	HCPMA-6.9 μm		HCPMA-10.3 μm		HCPMA-12.9 μm		HCPMA-14.9 μm		HCPMA-17.0 μm	
		Fine Gradation		Mid Gradation		Mid Gradation		Mid Gradation		Coarse Gradation	
(mm)	(m^2^/kg)	% Passing	SA (m^2^/kg)	% Passing	SA (m^2^/kg)	% Passing	SA (m^2^/kg)	% Passing	SA (m^2^/kg)	% Passing	SA (m^2^/kg)
19	0.41	99.9	0.41	99.9	0.41	99.9	0.41	99.9	0.41	99.9	0.41
12.5	-	92.3	-	92.3	-	92.3	-	92.3	-	92.3	-
9.5	-	65.1	-	65.0	-	65.0	-	65.0	-	65.0	-
4.75	0.41	19.0	0.08	18.7	0.08	18.7	0.08	18.7	0.08	18.4	0.08
2.36	0.82	13.6	0.11	12.1	0.10	12.1	0.10	12.1	0.10	10.2	0.08
1.18	1.64	11.5	0.19	9.3	0.15	9.3	0.15	9.3	0.15	7.3	0.12
0.6	2.87	10.0	0.29	7.2	0.21	7.2	0.21	7.2	0.21	5.2	0.15
0.3	6.14	8.8	0.54	5.6	0.35	5.6	0.35	5.6	0.35	3.7	0.22
0.15	12.29	8.4	1.03	5.0	0.62	5.0	0.62	5.0	0.62	3.0	0.37
0.075	32.77	7.9	2.62	4.9	1.64	4.9	1.64	4.9	1.64	2.5	0.83
∑SA (m^2^/kg)		5.27		3.54		3.54		3.54		2.27	
Pbe (%)		3.63		3.64		4.56		5.28		3.84	
FT (μm)		6.9		10.24		12.87		14.90		16.94	

**Table 4:** Optimal Film Thickness for Performance and Aging Durability in Various Test Methods.

Test Method	Optimal Film Thickness		
	Performance before Aging	Performance after Aging	Aging Durability
Cantabro test	12.9 µm	12.9 µm	14.9 µm
SCB cracking test	14.9 µm	12.9 µm	12.9 µm
SCB fatigue test	14.9 µm	12.9 µm	12.9 µm
HWTT test	-	14.9 µm	14.9 µm

## Data Availability

The data presented in this study are available on request from the corresponding author.
